# Research Progress of Spectral Imaging Techniques in Plant Phenotype Studies

**DOI:** 10.3390/plants13213088

**Published:** 2024-11-02

**Authors:** Qian Zhang, Rupeng Luan, Ming Wang, Jinmeng Zhang, Feng Yu, Yang Ping, Lin Qiu

**Affiliations:** Institute of Data Science and Agricultural Economics, Beijing Academy of Agriculture and Forestry Sciences, Beijing 100097, China; zhangqian@baafs.net.cn (Q.Z.); zhangjinmeng@baafs.net.cn (J.Z.); yufeng@baafs.net.cn (F.Y.); pingyang@baafs.net.cn (Y.P.); qiulin@baafs.net.cn (L.Q.)

**Keywords:** plant phenotype, spectral imaging, HSI, MSI, LiDAR

## Abstract

Spectral imaging technique has been widely applied in plant phenotype analysis to improve plant trait selection and genetic advantages. The latest developments and applications of various optical imaging techniques in plant phenotypes were reviewed, and their advantages and applicability were compared. X-ray computed tomography (X-ray CT) and light detection and ranging (LiDAR) are more suitable for the three-dimensional reconstruction of plant surfaces, tissues, and organs. Chlorophyll fluorescence imaging (ChlF) and thermal imaging (TI) can be used to measure the physiological phenotype characteristics of plants. Specific symptoms caused by nutrient deficiency can be detected by hyperspectral and multispectral imaging, LiDAR, and ChlF. Future plant phenotype research based on spectral imaging can be more closely integrated with plant physiological processes. It can more effectively support the research in related disciplines, such as metabolomics and genomics, and focus on micro-scale activities, such as oxygen transport and intercellular chlorophyll transmission.

## 1. Introduction

Plant phenotype is the quantitative description of the individual development, physiological characteristics, and biochemical characteristics of plants. The acquisition and analysis of plant phenotype data are important in irrigation management, disease prevention and control, breeding research, and yield increases [1]. High-throughput and precise phenotype acquisition greatly promote the screening of breeding materials and significantly improve breeding efficiency. There are significant differences in plant phenotypes, such as morphology, physiology, and biochemistry, between different plants at different growth stages. Moreover, the growth environment of plants under natural conditions is complex and poses significant challenges to plant phenotype analysis. Traditional plant phenotype observation is mainly conducted through the manual measurement of various phenotypic parameters and has the drawbacks of being time-consuming and labor-intensive and having large errors and strong subjectivity [2]. Rapid, batch, and repeatable measurements of plant phenotypes have become bottlenecks in the fields of breeding and precision cultivation.

Spectral imaging technique, which has the advantages of being non-destructive, high-throughput, reliable, and capable of real-time operation and repeatable measurement, has good potential in plant phenotype analysis. It is widely used in precision agriculture and breeding [3]. A variety of spectral sensors have been developed and applied in plant phenotype analysis and trait observation [4]. In this field, vegetation indices (VIs) [5], remote sensing [6], unmanned aerial vehicles (UAVs) [7], high-throughput plant phenotype platforms (HTPPs) [8], and machine learning [9] are currently research hotspots [10].

Previous studies have discussed the application of certain spectral imaging techniques in plant phenotypes, such as UAV [7], near-infrared (NIR) [11], remote sensing [9], and LiDAR [12], as well as their application in certain plants or certain phenotypes, such as grains [13], fruits [14], and drought stress [3]. The phenotype analysis plan in different scales or spectral imaging techniques is worth learning. This article reviews the research progress in plant phenotypes based on the spectral technique in Web of Science, which systematically compiles the application of spectral imaging in plant phenotype analysis on different scales based on natural light spectral bands. This will provide a basic and detailed reference and inspiration for future research.

## 2. Spectral Imaging

Spectral features are observable changes in phenotypic images caused by electromagnetic features of one or more pixels. This is caused by the emission or absorption of photons, whose energy corresponds to the difference between the initial and final states of the transition. Spectral features can indicate regions of interest, such as diseases or abiotic/biotic stresses [15]. Although the photosynthetically active radiation of plants covers only a 400–700 nm spectral range, however, plants can interact with light in the 350–2500 nm spectral range [8]. From short to long, the spectral bands can be divided into the following types based on wavelength: gamma-ray, X-ray, ultraviolet (UV), visible light (VL), infrared, microwave, and radio. The infrared is usually divided into four zones: NIR, short-wave infrared (SWIR), middle-wave infrared, and long-wave infrared.

**Figure 1:**
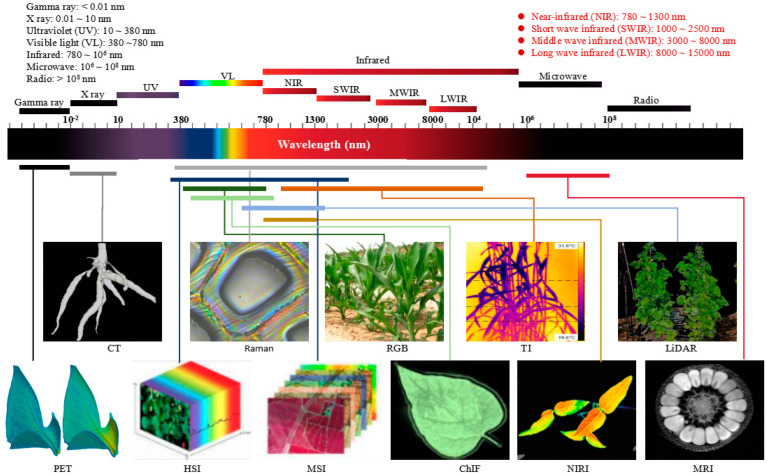
Spectral band division and various imaging techniques.

According to the frequency or wavelength used, electromagnetic waves can be reflected, absorbed, or transmitted through materials, which provides many potential quantitative quality characteristics. This non-destructive analysis technique uses the resolution of sensors and the mathematical econometric models [16]. Consequently, an array of optical sensors and sensing methods have been developed based on different electromagnetic responses at different wavelengths [4]. As represented in Figure 1, according to the order of spectral wavelengths from short to long, spectral imaging mainly includes the following types:

- Positron emission tomography (PET). This labels certain essential substances in plant metabolism (e.g., glucose, protein, and nucleic acid) with short-lived radioactive isotopes and reflects crop metabolic activity through the aggregation of these substances in metabolism activity [17].
- X-ray computed tomography (X-ray CT). This has a wavelength range of 10 pm–10 nm. It is used to detect the differences in energy absorption before and after scanning from different angles to visualize the external and internal three-dimensional (3D) structures of plants [18].
- Hyperspectral imaging (HSI). This has a wavelength range of 200–2500 nm. It is used to detect the two-dimensional geometric space and the one-dimensional spectral information of targets. It is based on a wide range of narrow-band image data and continuous and narrow-band image data with high spectral resolution [15].
- Multispectral imaging (MSI). This has a wavelength range of 200–2500 nm. It contains many discrete spectral bands that typically range from three to hundreds or a set of customized wavelength bands [10].
- Raman mapping (Raman). This has a wavelength range of 285–50000 nm. It is a scattering spectrum used to analyze the molecular structure and chemical composition of substances based on the Raman scattering effect [19].
- Visible imaging (VI). This has a wavelength range of 380–780 nm. It creates an RGB color image with three channels: red, green, and blue [20].
- Chlorophyll fluorescence imaging (ChlF). Although Chl fluorescence is emitted at around 600–750 nm, it can be excited in the 400 to 720–730 nm range. ChlF maps the emitted chlorophyll fluorescence signal to the sample space based on a pixel used to estimate photosynthetic performance and detect the effects of various stresses on plants [21].

- Light detection and ranging (LiDAR). This uses sensors to send light pulses to objects and receives reflected pulses from objects, and measures the distance between the object and the sensor based on the time required between transmission and reception. It can obtain parameters such as distance, orientation, altitude, velocity, attitude, and even the shape of the target [22].
- Near-infrared imaging (NIRI). This has a wavelength range of 780–1300 nm. It mainly records the infrared radiation reflected by objects [11].
- Thermal imaging (TI). This has a wavelength range of 1000–14,000 nm. The infrared radiation energy distribution pattern of the object being tested is received, and the obtained infrared thermal image is formed, which corresponds to the thermal distribution field on the surface of the object [23].
- Magnetic resonance imaging (MRI). This has a wavelength range of 1 mm–1 dm. The electromagnetic waves emitted by an object are detected by applying an external gradient magnetic field. The position and type of atomic nucleus of the object are detected, and an internal structure image is drawn [24].

There are slight differences in spectral band division among the different imaging methods in different research fields. There may also be intersections between different spectral bands; for example, MSI and HSI have covered VI, NIR, and SWIR.

## 3. Spectral Imaging Application in Plant Phenotypes

In this study, plants refer to the objects involved in agricultural production, such as grain crops, economic crops, feed crops, and green manure crops. Phenotype refers to external traits, such as shape, structure, size, and color, which are determined by genes, the environment, and related physiological and biochemical characteristics. Plant phenotype analysis is the study of various phenotypic information related to plant growth in complex environments, and it determines the structure, performance, and tolerance to limitations of individual plants or plant groups [15]. Plant phenotypes can be divided into four types: morphological phenotype, physiological phenotype, biochemical phenotype, and performance trait phenotype (Table 1).

The scattering and reflection, transmission, and absorption of photons are caused by the interaction between light and plant tissues involved in the propagation of light in plants. The spectral technique has been widely used in the phenotype analysis of crops, and sensors with different characteristics have different specific applications in agriculture [58,85,86,87]. Spectral data collection relies heavily on these sensors and affects the final phenotype results [88]. Different spectral imaging techniques can monitor changes in substances on different scales. PET and X-ray CT are used to detect the energy released by electron transitions in the inner shells of atoms. RGB and ChlF capture the energy changes caused by atomic valence electron transitions. HSI and MSI respond to energy changes caused by atomic electron and molecular vibrational transitions. TI, Raman, and MRI respond to molecular vibrational transitions. LiDAR generates precise 3D shapes by measuring variable distances (ranges) using laser pulses. NMR collects only spatial information, while infrared and Raman collect only spectral information [13]. The following system summarizes the application of spectral imaging techniques to plant phenotypes.

### 3.1. Morphological Phenotype

**Figure 2:**
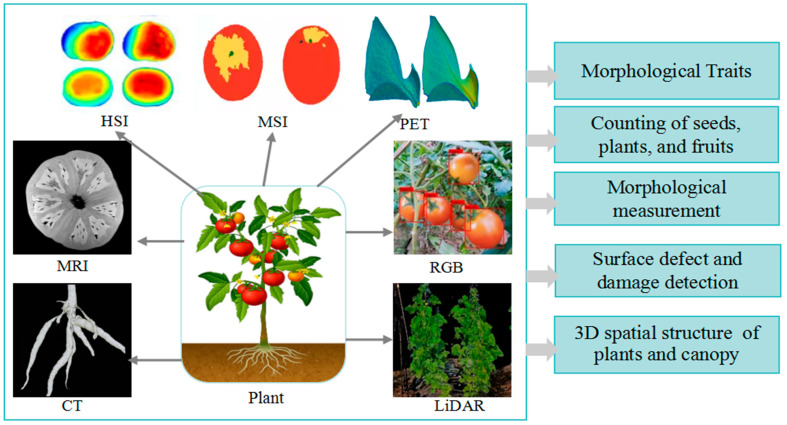
Application of spectral imaging in morphological phenotypes of plants.

The morphological phenotype is the most widely used in phenotype analysis because it is the basis for plant selection in breeding programs. Related research has covered almost all plant species. Its common research objects include seeds, fruits, leaves, flowers, plants, and canopies. Their common characteristics are shown in Table 1. Some unique morphological characteristics of specific crops have been proposed, such as the tiller number and main stem number for gramine crops [89,90], the sword leaf length and width for rice, and the number of grains and pods per plant for soybeans [91]. With the deepening and refinement of phenotype research, more unique phenotypic features will be proposed in the future. As represented in Figure 2, many spectral imaging techniques can be used to extract morphological traits.

HSI and MSI provide various types of spectral information related to plant physiological and biochemical properties, which can reflect the interaction between light, crops, and their biochemical components from cellular to landscape scales. MSI is considered a type of HSI. HSI and MSI can be used to detect and identify the leaf area index (LAI), diseases, and pests [69], as well as to classify plants, fruits, and seeds. Multi-temporal MSI based on UAV is used for detecting banana plants and individual plant counting [55]. MSI and HSI passive sensing are used to assess early plant vigor in winter wheat, and dry weight, nitrogen uptake, and nitrogen content are tested based on canopy cover images [89]. MSI is also used for the phenotypic analysis of ornamental greening plants’ leaf color changes and genetic analyses [92].

The RGB image is the most common form of VI. The combination of RGB images and a deep learning convolutional neural network (CNN) can achieve plant classification, fruit detection [32], and image segmentation [93]. It is widely used for variety classification and identification, weed and canopy segmentation [94], defect and germination detection, and growth stage and maturity evaluation [85,95]. RGB combined with CNN is used for apple 3D detection and location in orchards, and it has an F1-score of 0.881 [30]. It can also be used to obtain morphological parameters, such as color [79], LAI [78], canopy area, and other geometric attributes, and to detect plant leaves, tree crowns, and fruits. With the use of RGB imagery, an attention-based recurrent CNN has been proposed for accurate vegetable mapping from multi-temporal UAVs, and it has an overall accuracy of 92.80% [57].

LiDAR is a laser active sensing technique based on the time-of-flight principle, which generates high-density 3D point clouds through photon counting [12]. It can obtain the horizontal and vertical spatial structural characteristics of plants, as well as the plant height, biomass, and 3D distribution of the canopy. Plant growth and development can be examined by plant size and morphology. LiDAR can generate 3D points along the laser path to reveal some information below the canopy, which provides unique structural features for classifying crops at the canopy or patch level. LiDAR can be used for the construction of canopy 3D point cloud [22], extraction of plant height, biomass, size, and shape of various crops [96], assessment of plant growth and development, description of crop structure, shape, light interception [97], and response to irrigation [98], and detection of male spikes and lodging.

MRI captures and depicts detailed 3D images of anatomical structures by detecting the energy released when protons align with magnetic fields. MRI can provide spatial information for nuclear examination. It can visualize the structural and dimensional characteristics of the maize stem vascular system [99]. MRI can be used for examining the internal and anatomical characteristics of seeds, roots [100], internal damage to fruits, embryos, and endosperms, and for assessing crop quality and phenotype. It can also obtain 3D plant structures [101] and use them for seed growth in soil, and provide detailed images of plant and root structures [24]. MRI is also used for the early detection of verticillium wilt based on cotton roots [102].

X-ray CT can obtain detailed 3D structural information inside objects and is an excellent tool for 3D imaging and the quantitative analysis of plant tissues and organs [18]. Due to the density dependence of X-ray attenuation, it is particularly suitable for plant imaging, as the space between cells is ubiquitous in many plant organs [103]. X-ray CT can be used for extracting crop morphological traits, such as tillering, spike length, leaf veins, and intercellular spaces [18]; inspecting the internal and anatomical characteristics of fruits and seeds [104]; assessing the quality, maturity, and phenotype of fruits and seeds [105,106]; and for the 3D reconstruction of root systems [103].

### 3.2. Physiological Phenotype

**Figure 3:**
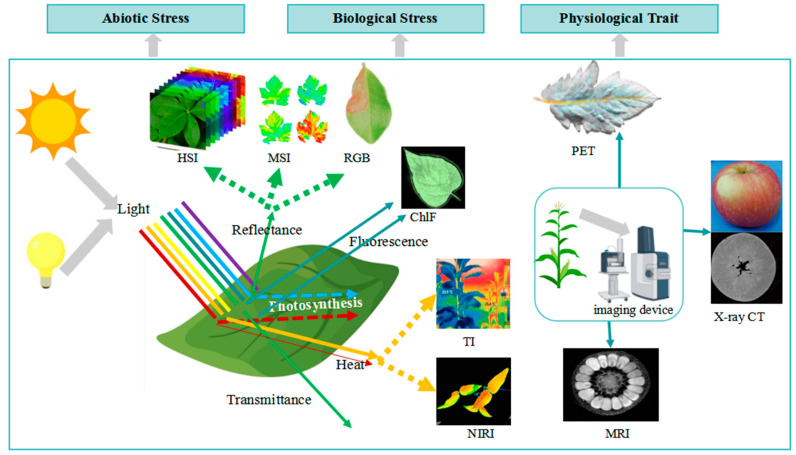
Application of spectral imaging in physiological phenotypes of plants.

The physiological phenotype is related to the physical state and function of plants, including their growth rate, reproductive ability, and stress resistance. These traits are usually examined with biochemical traits to elucidate the functions of cells, tissues, and organs. The basic principle of plant physiological phenotype analysis based on spectral technique is shown in Figure 3. Physiological traits are influenced by various factors, including temperature, light, moisture, and nutrition. Spectral imaging, combined with advanced machine learning (ML), has become the ideal tool for high-throughput crop physiological phenotype analysis [15]. Because physiological traits involve the functions of various parts of plants under specific environmental conditions, they are important in stress responses.

HSI and MSI can simultaneously measure the spectral characteristics of a wide range of continuous bands, which are usually used to explain the structural, chemical, and physical properties of plants. It has stronger phenotypic detection capabilities and can reflect plant performance and interactions with the environment in more detail [63]. Especially in the early stages of biotic or abiotic stress in plants, the naked eye or visible light cannot recognize the stress, while HSI and MSI can detect it [107]. Therefore, they have significant advantages in the early identification and monitoring of plant diseases and pests, as well as in stress response evaluation [69]. HSI can also be used for the identification of haploid polyploids and the prediction of seed and germination abilities [28]. MSI is widely applied in seed phenotyping and quality monitoring, such as physicochemical quality traits, defect detection, pest infestation, and seed health [16].

NIRI uses an electromagnetic wave located between VL and mid-infrared light and is widely used for the real-time physical and chemical analyses of plants [11]. NIRI uses infrared wavelengths that penetrate deeper than other instruments of the same wavelength, making it highly sensitive in identifying the presence of water, water stress, and the cellular physical structure. It can measure the function of swollen cell structures and perform the phenotypic analysis of roots in the dark. NIRI can be used to measure plant water distribution and drought stress, external defect detection [108], and quality control [109]. NIRI is also used to monitor invasive insect pests (e.g., brown marmorated stink bugs) on different vegetal backgrounds [72].

TI measures the surface temperature of plants by detecting their infrared radiation and can generate analysis data based on time series or single time points. As it can monitor subtle changes in plant canopy temperature at different growth stages and its response to the environment, it is used to select genes and assist in breeding by comparing the differences in leaf or canopy temperatures. TI is mainly used for estimating plant drought stress and transpiration [23] and moisture condition index calculation, including the crop water stress index (CWSI) and stomatal conductance indices [107]. It is also used for the quantification of crop osmotic stress response to salinity and the detection and monitoring of diseases and pathogen infections [110]. TI can assess the water status of a wide range of individuals and is used to test the water stress of apples [111].

ChlF can reflect the spatial and temporal heterogeneity of fluorescence spectra on multiple scales, such as cells, leaves, and plants, thus making it the most accurate and appropriate method for screening the effects of environmental stress on plants. It can monitor plant metabolic information, detect plant diseases, and invert chlorophyll and nitrogen content, nitrogen–carbon ratio, and LAI. Non-modulated ChlF can explore in depth the intrinsic photosynthetic physiological information of plants. ChlF is mainly used for detecting biological or abiotic stresses related to photosynthesis [75] and for analyzing the nutritional and physiological status of plants [112], such as photosynthetic capacity, non-photochemical quenching, and other physiological characteristics. It obtains the photosynthetic activity of crops in real time. ChlF and MSI are used to monitor the drought stress of common beans [21].

PET is a nuclear imaging technique that can generate 3D images or images of functional processes [17]. It can perform non-invasive imaging of the distribution of biomarkers, evaluate processes at the molecular and cellular levels, detect plant stress [113], and provide quantitative data in a non-destructive and dynamic manner [114]. A novel design of dedicated plant PET scanners specifically developed to address agronomic issues has been proposed [17].

Other spectroscopic techniques also have many applications. RGB is mainly used for pests and disease detection and identification, as well as quality evaluation and grading [26]. MRI is mainly used for detecting plants’ germination ability, dormancy, survival, vitality, and pest infestation [100], as well as the water status and transportation in plant cells [99]. X-ray CT is mainly used for quantifying the degree of drought or salt stress and detecting germination ability, dormancy, survival, vitality, and pest infestation [115,116].

### 3.3. Biochemical Phenotype

**Figure 4:**
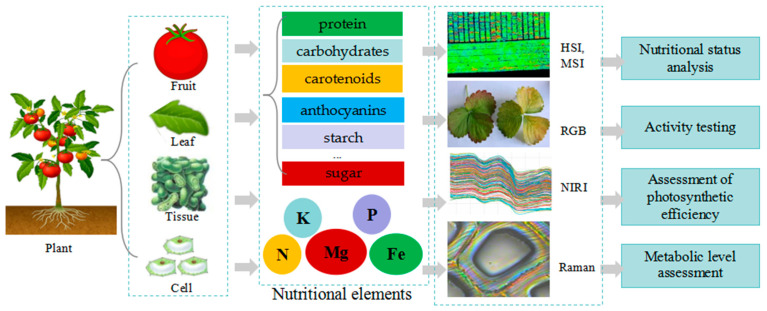
Application of spectral imaging in biochemical phenotypes of plants.

The biochemical phenotype characterizes the presence, composition, and quantity of specific chemical and biochemical markers under steady-state conditions. These traits are related to various aspects of biological processes, such as leaf nitrogen, protein, carbohydrates, carotenoids, fatty acids, and chlorophyll content involved in photosynthesis, metabolism, and hydraulics (Figure 4). Photosynthetic pigments are important indicators of plants’ photosynthesis. Chlorophyll [64] and carotenoids can be evaluated for photosynthetic pigments at the leaf and canopy levels using RGB, HSI, ChlF, and NIR. The use of spectroscopic techniques to observe and identify targeted biochemical markers has promoted various omics and breeding research. Early and long-term nutrient deficiencies, such as nitrogen, phosphorus, potassium, magnesium, and iron, can be monitored using ChlF and MSI.

HSI and MSI are usually divided into three regions: the VIS region shows strong absorption of photosynthetic pigments, lutein, chlorophyll, and carotenoids; the VIS-SWIR region can extract information on general nutrients, such as protein, nitrogen, and sugar; the NIR and SWIR regions are sensitive to water and nitrogen content; and the SWIR area quantifies plant characteristics, such as phosphorus, hemicellulose, protein, and mineral content. HSI can compare the changes in special substances in plant bodies and show the reflectance of leaf sponge tissue, leaf biochemical components, and the main vegetation index of the canopy. HSI and MSI can be used to examine water content, plant nutrients, canopy chlorophyll content, nitrogen content, many VIs, and other biochemical parameters. HSI is used to characterize cotton photosynthesis at the canopy level, with a suitable spatial resolution and scanning throughput at the canopy and sub-canopy levels [63]. HSI and RGB images are used for the high-throughput analysis of leaf chlorophyll content in hydroponic lettuce [64].

NIRI relies on the absorption of NIR radiation by components, such as proteins, lipids, carbohydrates, and water, which can generate unique spectral patterns. It can detect related energy absorption and the molecular vibrations of the combination of the C-H, N-H, and O-H functional groups [16]. NIRI can be used for non-destructive testing and variety identification of agricultural products [108,117] and has been used to detect and visualize the distribution of sugar content in agricultural products in over 1,000 packaging factories in Japan. NIRI is also used for evaluating fruit maturity and hardness, quality and nutritional analysis, and the detection of survival and vitality.

ChlF comes with a standard filter wheel that can achieve multispectral fluorescence imaging. Fluorescence imaging is mainly used to measure the optical properties of chlorophyll. ChlF is commonly applied to assess the spatial patterns, photosynthesis, and metabolic status of crops. It can effectively inverse chlorophyll, nitrogen content, and nitrogen carbon ratio, detect plant metabolic information and diseases, and analyze the vertical heterogeneity of canopy biochemical parameters [118]. Chlf and HSI are used to determine shikimic acid concentrations in transgenic maize exhibiting glyphosate tolerance, which provides a new data-driven method.

Raman is a scattering spectrum that exposes a sample to the spectrum and measures the degree of light scattering caused by molecular bond vibrational transitions. It is commonly used in molecular structure research. As a non-invasive technique, it is popularly used in biochemical and structural analyses, which provide insights into the structure, concentration, and interaction of biochemical molecules within an organism’s cells and tissues [119,120]. Raman is mainly used for the analysis of plant biochemistry and structure; the detection of biochemical molecules, cells, and tissues [121]; the assessment of plants’ nutritional content [122]; and disease detection [123,124].

### 3.4. Performance Phenotype

**Figure 5:**
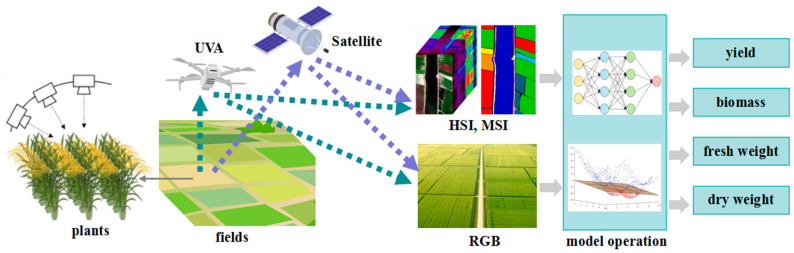
Application of spectral imaging in performance phenotype of plants.

The performance phenotype describes the overall performance of crops in terms of biomass, yield, and quality, and it is the most complex but interesting trait for crop breeders. The basic principle of plant performance phenotype analysis based on spectral technique is shown in Figure 5. Its common parameters include harvest index, number of grains per spike, thousand-grain weight, number of grains per plant, hundred-grain weight, number of spikes per mu, and theoretical yield. Although the feasibility and accuracy of the performance phenotype have been proven, its stability and repeatability cannot be strictly guaranteed. Due to complex factors, such as genotype, environmental factors, and agronomic practices, performance phenotype is influenced by a combination of factors.

HSI and MSI can be used for biomass and yield prediction and growth stage and maturity evaluation. HSI is used to train a CNN classification model to estimate corn grain yield, with a classification accuracy of 75.50% at five corn growth stages [61]. RGB and depth images are used to estimate various growth indices of four varieties of greenhouse lettuce, and the normalized root mean square error of fresh weight is 6.09% [85]. Researchers [80] have proposed a CNN approach using UAV-RGB imaging to estimate dry matter yield traits in a guinea grass breeding program.

Other spectral imaging techniques can be applied to crop phenotype research, such as synthetic aperture radar (SAR) and laser backscatter imaging (LLBI). SAR can work in weather conditions with very low visibility (e.g., cloud cover). It has been widely explored in crop classification, crop growth monitoring, and soil moisture monitoring [125]. LLBI is a low-cost imaging technique that utilizes the principles of light absorption, scattering, and image processing in visible and NIR electromagnetic spectra to detect and analyze targets [126].

## 4. Comparative Analysis of Spectral Imaging

### 4.1. Spectral Imaging Comparison

**Figure 6:**
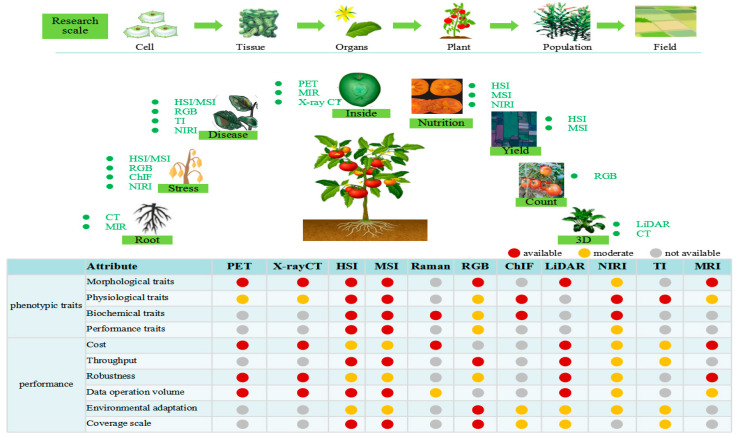
Application of spectral imaging to crop phenotypes.

Different spectral imaging techniques are suitable for different crop phenotype analysis tasks, as shown in Figure 6. X-ray CT and LiDAR can measure the 3D morphological features of crops, and they are appropriate for the 3D representation of crop surfaces, tissues, and organs. Specific symptoms caused by plant nutrient deficiency can be easily detected using HSI, MSI, LiDAR, and ChlF measurements. HSI, IR, and Raman provide multi-component information that can overcome the low sensitivity. In addition, HSI and Raman are highly sensitive to the detection of trace components. ChlF surpasses HSI in characterizing photosynthetic activity on the micro-scale. Table 2 compares the different imaging techniques.

Crop phenotype analysis can be divided into different scale levels: cell and tissue level, organ level (root, stem, leaf, flower, fruit, seed, and harvest), plant level, and population and plot level. X-ray CT and MRI can provide anatomical details of crop tissues, while Raman can visualize cell walls, requiring the use of magnified images, such as microscopes. ChlF is used to examine the function and nutritional information of seeds. NIR effectively evaluates the internal quality attributes of fruits in a non-contact manner. LiDAR can show the 3D spatial structure of leaves and plants. The MSI phenotype has potential in the genetic analysis of seasonal leaf color changes in green crops [92]. HSI, MSI, and RGB are suitable for large-scale, high-throughput crop phenotype analysis that can be combined with agricultural machinery, drones, satellites, and other devices.

### 4.2. High-Throughput Plant Phenotyping Platform (HTPP)

HTPP applies modern information techniques for the quick, automatic, and non-destructive acquisition, analysis, and in situ monitoring of complex plant traits [128], thus making it possible to simultaneously measure many plant traits [112]. HTPP typically uses multiple sensors to measure various traits of crops, determine nutrient, water, and pesticide requirements, and detect various biological stresses. It is usually divided into two types: outdoor and indoor.

Outdoor HTPP is typically conducted on farms or in natural ecosystems using only natural light sources. Spectral sensing equipment is always installed in agricultural machinery, drones, fixed-wing aircraft, vehicles, and satellites for large-scale phenotype analysis. Aerial platforms provide remote sensing techniques for monitoring crop growth and various stresses and estimating large-scale crop yields [110]. In recent years, the HTPP for unmanned aerial vehicles equipped with multiple sensors has provided a large-scale, efficient, non-invasive, flexible, and low-cost solution for large-scale breeding [50,129,130]. It still faces many challenges, such as cross-platform data acquisition, sensor calibration, data processing methods [131], image interpretation, and the reliable and accurate extraction of crop phenotype information [132].

Indoor HTPP includes greenhouse, growth chamber, and laboratory scenarios. Crops can be fully or partially illuminated by artificial light sources. Typically, automated systems and handheld devices are used for imaging, which is suitable for a limited number of crops. Phenotypic analysis includes plant growth rate, crop stress detection, biomass estimation, seed viability, root characteristics, and physiological and biochemical measurements. Portable devices and their isolation measurement rooms are widely used. Handheld devices are simple, easy to use, and cost-effective, but they have small coverage and slow measurement speed and are labor-intensive and time-consuming.

## 5. Research Trends

### 5.1. Multimodal Data Application

Multimodal data application is a future research trend. Plants’ TI and MSI multimodal outputs can be compared and analyzed to provide complementary insights and to effectively develop VIs, which can provide new methods for plant stress physiological responses. Software has been developed for calculating CWSI and the green–red nutritional index by fusing NIR and RGB images. Multimodal data can improve existing yield prediction models, such as VIs, weather, soil, number of fruits/flowers, and canopy height. TI combined with RGB can accurately segment crop images of different VIs [133]. There is still room for the fusion and application of various types of remote sensing images, and combining a large amount of physical and spectral data with biochemical data on crop growth has great value [134].

### 5.2. 3D Image Application

Spatial 3D reconstruction of crops is important for high-throughput crop phenotype acquisition, plant type feature evaluation, and phenotype correlation analysis. Due to the limitations of physical space in the transport of water, gas, and nutrients in living organisms, the 3D analysis of plant structures is of great significance. Many physiological processes of crops, such as photosynthesis, respiration, and growth, are controlled by the transportation of water, metabolic gases, and nutrients, which are essentially 3D. The 3D structure research of crop phenotypes mainly includes the spatial structure of individual plants or populations, stem vein network, nutrient spatial transport mechanism, plant posture estimation, monitoring of lodging, leaf orientation distribution, the spatial structure of leaf sequence, inflorescence, and fruit sequence. The 3D imaging of crop organs and tissues is helpful for understanding their role in water, gas, and nutrient transport. The 3D images obtained from X-ray CT can be used for the quantitative modeling of crop physiological processes, precise numerical calculations of heat and mass transfer in crops, and other biophysical processes. By combining the realistic 3D arrangement of cells and tissues within plant organs, complex phenomena can be analyzed more accurately through simulations. For example, in respiration and photosynthesis, the connectivity of related transport structures can be studied using networks of pores or vascular systems.

### 5.3. Micro-Scale Applications

Spectral phenotype analysis techniques increasingly need to be extended to the substructure level, and imaging techniques should continuously surpass the current physical spectral level. Spectral techniques have been applied to many microscopic phenotypic studies, such as pollen, stomata, maize vascular bundles, mesoporous structures in fruit tissues, and cell stress. X-ray CT has opened up possibilities for the high-throughput phenotype analysis of plant organs [18]. For example, it can visualize the fluid and solute transport structures in plant organs; examine the structural morphology of the xylem in tomato roots and the cellular structure of the vascular system in tomato petioles; separate different tissues, such as apple ovaries, from 3D images; and separate cells from pore spaces on the microscopic scale. The rapid development of spectral imaging techniques is helping to connect genotype–phenotype differences. Although research on phenotypes on the micro-scale is currently relatively insufficient, it is an inevitable trend for future development.

### 5.4. Low-Cost Portable Imaging Device

The most important aspect of the crop phenotype analysis platform is its ease of use and affordability. The miniaturization of optical sensors can further promote the improvement of their performance and the flexibility of integration with different phenotype platforms. Breeding works require simple phenotypic tools, such as collecting wheat canopy reflectance data through handheld devices [135]. The handheld NDVI measuring instrument is simple, practical to use, and low cost, and it can identify plant vitality and biomass. The main characteristics of future phenotype devices included a low level of professional knowledge, simple application operation and data input, multiple sensors integrated into a portable device, and the strong stability and robustness of algorithms in complex environments.

### 5.5. Application of Machine Learning

ML is the future development direction of multi-omics, data integration, and systems biology [59]. Linking piled-up genomic information with trait expression still faces challenges, and remote sensing and ML can address the association between massive genomic information and trait expression in the future [52]. By combining computer science, biology, remote sensing, statistics, and genomics, ML can associate complex plant traits with gene expression in the future. ML has found increasing application in the processing of HSI data due to its nonparametric nature and strong flexibility in dealing with the nonlinear relationship between hyperspectral reflectance and target parameters. ML can already accurately predict the biochemical pathways of tomatoes using metabolite data [136]. The future ML platform must be robust, flexible, and able to distinguish multiple disease symptoms on a single leaf or the same plant canopy. Automated machine learning is an automated version of ML for dealing with large and complex multivariate datasets. This automation saves time and effort and enhances model quality, which is the future development trend.

## 6. Conclusion and Outlook

This article systematically analyzes research progress in spectral imaging techniques in crop phenotype analysis. Currently, there is insufficient research on physiological and biochemical phenotypes, especially biochemical phenotypes. There is abundant research on the macro-scale, especially on the field remote-sensing scale, unmanned aerial vehicles, and vehicle-mounted devices, while there is relatively little research on the micro-scale, especially on the substructural levels of molecules, cells, and organs. Future crop phenotype research based on spectral imaging should be more closely integrated with plant physiological processes, focusing on oxygen transport, intercellular chlorophyll transmission, and other micro-scale activity, and support research on related disciplines, such as metabolomics and genomics, more effectively.

Currently, the greatest challenge in phenotype research is quickly obtaining high-dimensional, high-density, and high-precision, large-scale plant phenotype data from individual molecules of the entire organism. How to effectively define and extract complex traits and how to improve accuracy and throughput remain key issues. HSI and MSI still have great development potential, which could be further developed in areas such as multi-device collaboration, spectral fusion, airborne equipment improvement, and real-time image processing techniques. The potential of sensors in obtaining new phenotypic information still needs to be explored, and new sensors and sensing methods for complex traits should be studied. Spectral imaging techniques should be improved in hardware modes, image reconstruction and analysis, resolution and contrast enhancement, mathematical modeling, and data sharing. Integrating a universal metabolomics platform would be ideal, and whole-plant physicochemical phenotype analysis would be the next key goal.

## Figures and Tables

**Table 1:** Main characteristics of plant phenotype monitoring by spectral technique.

	Research Object	Phenotypic Characteristics	Imaging Techniques	References
Morphological Phenotype	Seed	size, shape, quantity, incompleteness	HSI, MSI, RGB, PET, MRI, X-ray CT	soybeans [16], rice [25], hazelnut [26], pepper seed [27], hybrid okra seed [28], chickpea [29]
	Fruit/Ear	size, shape, quantity, color, ear length, ear thickness, symmetry, incompleteness, maturity	HSI, MSI, RGB, PET, X-ray CT	apple [30], sorghum panicle [31], kiwifruit [32], tomato [33], litchi [34], grape [35]
	Leaves	leaf area, length, width, number, inclination angle, color, veins, texture, symmetry	HSI, MSI, RGB, PET	leaves [36], grape [37], mango [38], sweet potato [39], tomato [40]
	Flower	quantity, color, number of petals, degree of openness	HSI, MSI, RGB	cool-season crops [41], apple [42], coffee [43], strawberry [44], flower [45]
	Root	root morphology, tuberous roots, lesions, defects	MRI, X-ray CT	potato [46], carrot [47], root [48], sorghum [49]
	Plant	plant height, stem thickness, leaves number, leaf area ratio, canopy coverage, stem length, plant spacing	HSI, MSI, RGB, LiDAR	maize [50], tree seedling [51], rice seedling [52], lettuce [53], oilseed [54], banana [55]
	Canopy	biomass, canopy coverage, coverage rate, average leaf angle, 3D spatial structure	HSI, MSI, RGB, LiDAR	tomato [56], vegetables [57], oil palm tree [58], rice [59], blueberries [60], corn [61]
Physiological Phenotype	Plant	leaf texture, leaf surface temperature, photosynthetic capacity, seed hardness, fruit hardness, canopy temperature, texture, density	HSI, MSI, RGB, TI, MRI, X-ray CT	tomato [62], cotton [63], lettuce [64], cucumber [19]
	Biological Stress	disease stress, disease spots, disease severity, pest stress, weed stress	HSI, MSI, RGB, NIRI, MRI, X-ray CT	mango [38], weed [65,66,67,68], grape [69], wheat [70], oats [71], plants [72]
	Abiotic Stress	drought stress, high/low-temperature stress, salt stress, nutritional stress	HSI, MSI, ChlF, NIRI, TI, PET, MRI, X-ray CT	common bean [21], watermelon [73], tomato [74], basil [75], grape [76]
Biochemical Phenotype	Plant	protein, carbohydrates, nitrogen content, carotenoids, fatty acids, chlorophyll content, water content, anthocyanins, starch, sugar	HSI, MSI, RGB, ChlF, NIRI, Raman	sorghum [77], sugar beet [78], corn [79]
Performance Phenotype	Plant	yield, quality, biomass, fresh weight, dry weight	HSI, MSI, RGB, NIRI	guinea grass [80], kiwifruit [81], corn [61,82], wheat [83], soybean [84]

**Table 2:** Comparison of different spectral imaging techniques.

Techniques	Advantages	Disadvantages
HSI, MSI	non-invasive, fast, and high-throughput; MSI provides higher spatial resolution than HSI; capture stress signals before visible	high cost and heavy weight compared to RGB sensors; high data dimension requires greater computing power, time, and resources; unsuitable for online applications [95]; limitations on plant research at small-scale or patch level [127]; higher challenges for data mining and machine learning
RGB	qualitative, reliable, inexpensive, convenient, and wild used; advantages in spatial resolution, signal-to-noise ratio, throughput, and repeatability	limited image accuracy due to inherent size distortion between 2D planes and 3D plants; only obtain surface features due to inability to penetrate the crop canopy; unsuitable complex environments with variable lighting, observation angles, object directions, and various occlusion [9]
NIRI	penetrates deeper than other instruments of the same wavelength; highly sensitive in identifying water’s presence, water stress, and cellular physical structure	unable to provide reliable data on plant chemical composition; relatively large relative error due to the interference between adjacent peaks in the spectrum; dependent on mathematical model to conduct analysis; sensitive to temperature and humidity
TI	monitors plant stress responses simpler and cheaper; higher spatial resolution, targeting, and sensitivity to certain environmental factors in a constantly changing environment	only obtains features related to surface temperature; relatively poor spatial resolution and repeatability; higher cost and more difficult to deploy compared with infrared thermometers; very limited effectiveness in small temperature differences [76]; easy to be disturbed for soil, air, and canopy temperature
LiDAR	durability, high accuracy, data resolution, and reading speed, and sensitivity to small distance changes; suitable for various lighting conditions, such as nighttime and field measurements	high cost, large data volume, and narrow band; unsuitable for complex leaf angles and flat canopy; time-consuming and large computational load for 3D point cloud generation; scanning noise was easily generated due to wind and rain interference; low accuracy in large-scale phenotype analysis; difficult analysis
ChlF	change in ChlF can occur before most other signs of stress; fast, non-invasive, easy-to-operate, low-cost, and highly sensitive; short measurement time, large measurement area, and high flux	vulnerable to interference from uneven lighting, wind, and rain; unable to distinguish potential causes of the stress; difficult to distinguish temperature signals and light signals when outdoors; unable to measure soluble solids content, fruit pH value, and maturity of the plant; requires dark-adapted measurements
MRI	provides spatial information of the nucleus; suitable for obtaining plant morphological characteristics under limited flux and spatial resolution	only operated in laboratory; no suitable portable devices for the in-field crop; long time consumption of data collection and limited throughput; unable to be used on aerial platforms due to the size and weight of the equipment; very high cost
X-ray CT	high spatial resolution, signal-to-noise ratio, and repeatability; multi-spectral X-ray provides higher sensitivity for plant identification	high cost, long time consumption, and low throughput; poor environmental adaptability; not suitable for airborne use; only scans roots with a diameter of 1 mm or more; unable to measure many fine roots; only achieves 3D visualization and qualitative interpretation of plant organs and tissues; low automation
Raman	high spectral resolution; highly sensitive for the detection of minor components; surface-enhanced Raman has better sensitivity	risk of tissue burns when laser irradiation applied due to small sample volume and self-luminous high; very weak and unstable—should be combined with other methods; strong interference of biological fluorescence signals in the background; high cost

## Data Availability

The original contributions presented in the study are included in the article. Further inquiries can be directed to the corresponding author.
